# A high throughput screen for next-generation leads targeting malaria parasite transmission

**DOI:** 10.1038/s41467-018-05777-2

**Published:** 2018-09-18

**Authors:** Michael J. Delves, Celia Miguel-Blanco, Holly Matthews, Irene Molina, Andrea Ruecker, Sabrina Yahiya, Ursula Straschil, Matthew Abraham, María Luisa León, Oliver J. Fischer, Ainoa Rueda-Zubiaurre, Jochen R. Brandt, Álvaro Cortés, Anna Barnard, Matthew J. Fuchter, Félix Calderón, Elizabeth A. Winzeler, Robert E. Sinden, Esperanza Herreros, Francisco J. Gamo, Jake Baum

**Affiliations:** 1 Department of Life Sciences, Imperial College London, Sir Alexander Fleming Building, Exhibition Road, South Kensington, London SW7 2AZ UK; 2 Diseases of the Developing World (DDW), GlaxoSmithKline, 28760 Tres Cantos, Madrid Spain; 3 School of Medicine, University of California San Diego, 9500 Gilman Drive 0760, La Jolla, CA 92093 USA; 4 Department of Chemistry, Imperial College London, Exhibition Road, South Kensington, London SW7 2AZ UK

## Abstract

Spread of parasite resistance to artemisinin threatens current frontline antimalarial therapies, highlighting the need for new drugs with alternative modes of action. Since only 0.2–1% of asexual parasites differentiate into sexual, transmission-competent forms, targeting this natural bottleneck provides a tangible route to interrupt disease transmission and mitigate resistance selection. Here we present a high-throughput screen of gametogenesis against a ~70,000 compound diversity library, identifying seventeen drug-like molecules that target transmission. Hit molecules possess varied activity profiles including male-specific, dual acting male–female and dual-asexual-sexual, with one promising *N*-((4-hydroxychroman-4-yl)methyl)-sulphonamide scaffold found to have sub-micromolar activity in vitro and in vivo efficacy. Development of leads with modes of action focussed on the sexual stages of malaria parasite development provide a previously unexplored base from which future therapeutics can be developed, capable of preventing parasite transmission through the population.

## Abstract (web summary)

Sexual forms of malaria parasites are responsible for transmission to the mosquito. Anti-malarial drug resistance remains a serious problem and requires advent of new drug therapies. Here, the authors present a high-throughput screen of potential antimalarial compounds, identifying seventeen drug-like molecules specifically targeting transmission.

## Introduction

Success in reducing the burden of malaria disease since the turn of the millennium has seen the paradigm shift from control to one of regional elimination and eventual global eradication^1,2^. The coordinated roll-out of established measures has yielded substantial reductions in mortality and morbidity and helped to achieve elimination of malaria in 10 countries^3^. However, to ensure that these gains are maintained and accelerated, and in light of worrying emergence of multidrug-resistant malaria in Southeast Asia^4^, new and innovative therapeutics are needed to prevent a potential reversal in numbers in the coming years.

At the heart of malaria disease incidence is spread of the *Plasmodium* parasite in the population via an infected *Anopheles* mosquito bite. Fundamental to this process is a switch in parasite biology from asexual replication to sexual development. Approximately 0.2–1% of asexual parasites undergo this alternative sexual developmental pathway to form mosquito-infectious male and female gametocytes^5^. Gametocytogenesis in *Plasmodium falciparum*, the species most deadly to humans, takes place over 10–12 days with the developing gametocytes, initially sequestered in the bone marrow, re-entering peripheral blood upon reaching maturity^6^. Mature gametocytes then maintain an arrested state awaiting uptake into the mosquito upon blood feeding. The consequence of this comparatively quiescent state is an insensitivity to most current antimalarials^7^ leading to a situation whereby a patient can be readily cured of malarial symptoms (killing asexuals) but remain infectious to mosquitoes and as such able to perpetuate disease spread. Successful transmission itself requires uptake of both male and female gametocytes into the mosquito midgut, which immediately sense a change in local environment and rapidly differentiate into male and female gametes. Male gametogenesis involves three rapid rounds of endomitosis and assembly of eight motile, flagellated sperm-like gametes^8^. Female gametogenesis involves expression of translationally repressed mRNA stored at the gametocyte stage^9^. Fertilisation ensues in the midgut forming a motile zygote that migrates to and through the midgut epithelium before forming an oocyst, establishing an infected mosquito state. Transmission is a huge population bottleneck in the parasite life cycle with natural mosquito infections usually having only ~2–5 oocysts (compared to 10^11^ asexual parasites in an infected host)^10^.

The concept of targeting sexual stages specifically, as a strategy to reduce malaria incidence, has received increased interest recently^11^. This follows emerging (re)appreciation that targeting the malaria parasite at the acute population bottleneck of transmission has potential benefits over asexually targeted therapeutics both as a means to break the cycle of infection but critically also as a barrier to the spread of drug-resistance alleles^11,12^. Conceptually, evolution of resistance to drugs specifically targeting the transmission stages will take far longer to develop than for conventional antimalarials, as selective pressure is only applied to the relatively small (non-replicating) gametocyte population compared to drugs targeting the ~10^11^ asexual parasites constantly replicating within a malarial host. Whilst quiescent in the human host, stage V gametocytes are poised for rapid onward development immediately upon mosquito blood-feeding, utilising distinct pathways to achieve their sex-specific roles during gametogenesis and fertilisation. Despite these observations, most current gametocyte assays utilise only an indicator of the metabolic viability of the gametocyte to determine compound activity and/or do not distinguish between male or female gametocytes e.g. mitotracker^7,13^, luminescence^14,15^, metabolic activity^16,17^ or are limited to female gametogenesis^18^. In contrast, the *P. falciparum* Dual Gamete Formation Assay (Pf DGFA) uses male and female gamete formation as sensitive reporters of the functional viability (i.e. their ability to undergo further development) of both gametocytes individually and is highly predictive of transmission-blocking activity in the current laboratory gold standard transmission assay—the standard membrane-feeding assay (SMFA)^19^. Finally, whilst high-throughput screening (HTS) of millions of compounds in pharmaceutical libraries has populated the antimalarial development pipeline in the past decade, with promising new chemotypes targeting the disease-causing asexual stages^20–22^, drug activity against other parasite stages have primarily only been considered when this property added extra value to existing identified molecules rather than essential properties in their own right. As such, very few studies have screened for sexual stage-specific activity without pre-filtering on asexual activity first^7^.

Here we have undertaken HTS of a large unbiased chemical diversity library where the primary filter for hit identification is specifically the ability to target transmission itself, using the Pf DGFA. Profiling of selected hits revealed a diverse range of activities both dependent and independent of asexual activity and some showing gametocyte sex-specific activity. We show that exemplar molecules from each activity class appear to inhibit transmission by different mechanisms and that this directly translates into a blockade of mosquito transmission. In particular, we identify a novel *N*-((4-hydroxychroman-4-yl) methyl) sulphonamide (N-4HCS) scaffold that rapidly inhibits the process of male gamete formation in vitro and blocks transmission in vivo in a mouse model of malaria. The N-4HCS scaffold shows excellent chemical tractability, favourable physicochemical properties and promise for further development as a transmission-specific therapeutic that could be used in new antimalarial combination therapies.

## Results

### Gametocyte and asexual-targeted compounds within the GHCDL

**Fig. 1:**
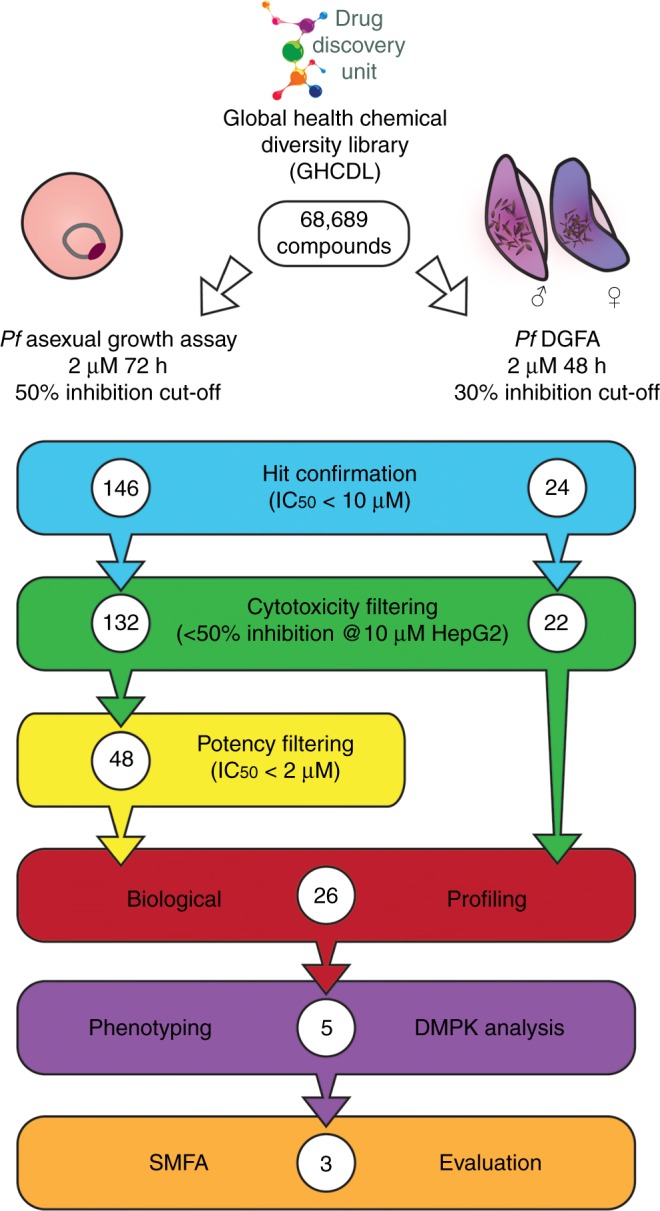
Screening progression cascade. The Global Health Chemical Diversity Library (GHCDL) was screened in parallel against *Plasmodium falciparum* asexual stage and male and female gametocyte. Actives were reconfirmed and cytotoxic compounds removed. Asexual hits were further filtered based upon potency and 26 compounds selected for further profiling based upon potency and commercial availability. Five compounds with different transmission-blocking properties were further investigated for activity phenotype against male gametogenesis and their physiochemical properties (DMPK) investigated. Activity of three molecules was confirmed by standard membrane-feeding assay (SMFA)

The Global Health Chemical Diversity Library (GHCDL) comprises 68,689 compounds dissimilar to molecules already known to have antimalarial properties. All molecules possess lead-like physicochemical properties and are arranged into structurally similar clusters of between 3 and 14 compounds. The Pf DGFA measures the functional viability of Stage V male and female gametocytes as reported by their ability to form male and female gametes^23^. The GHCDL was screened in the Pf DGFA at 2 µM with a 48 h incubation prior to triggering gamete formation, yielding 24 hits with an IC_50_ < 10 µM (19 male-specific, 3 female-specific, 2 dual-active) (Fig. 1). Of these, 22 hits showed <50% inhibition at 10 µM against human HepG2 cells (i.e. are non-toxic) (Supplementary Data 1). Of note, six of the male-specific compounds possessed a similar N-4HCS core structure.

In parallel the GHCDL was screened against asexual blood stages at 2 µM over a 72 h incubation using lactate dehydrogenase activity as a surrogate readout for parasite growth. Screening yielded 146 hits with an IC_50_ < 10 µM; 132 of which showed <50% inhibition at 10 µM against human HepG2 cells (chemically classified in 16 clusters and 97 singletons) (Supplementary Data 2). For prioritisation, only asexual-active compounds with an IC_50_ < 2 µM were progressed for downstream characterisation (48 in total).

### Multistage biological profiling of hit compounds

**Fig. 2:**
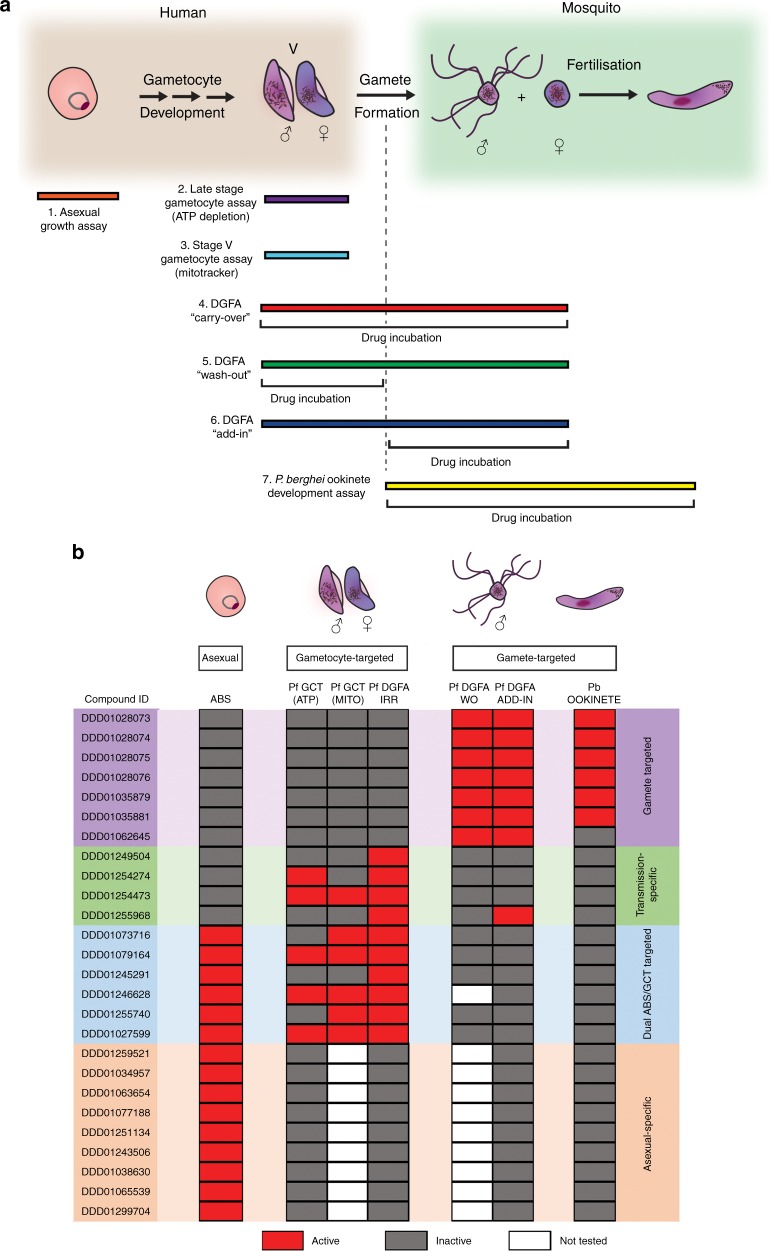
Profiling the transmission blocking properties of selected compounds. **a** 26 compounds were profiled in seven assays that interrogate different ranges of parasite cell biology (represented by position and length of coloured bars). Ability to prevent asexual replication was tested by asexual growth assay^20^. Ability to inhibit the metabolic viability of stage IV/V gametocytes (Pf GCT) was tested by late stage gametocyte ATP depletion assay (ATP)^43^ and stage V gametocyte by mitotracker assay (MITO)^7^. By varying compound incubation period, the carry-over/wash-out (WO)/add-in Pf DGFA assays permit discrimination between gametocyte- and gamete-targeted activity^23^. The *P. berghei* ookinete development assay^37^ tests activity against early mosquito stage parasites and cross-reactivity to *P. berghei*. **b** Table summarising outcome of compound profiling. Compounds fell into four categories: gamete-targeted (purple); transmission-specific (gametocyte-targeted, green); dual asexual/gametocyte targeted (blue); asexual-specific (orange). Red blocks indicate compound was active in respective assay (IC_50_ > highest assay concentration: 12.5 µM asexual; 10 µM ATP; 25 µM MITO; 12.5 µM WO; 10 µM Add-in; >12.5 µM Pb Ookinete), grey indicates inactive and white indicates not tested. The exception being the Pf DGFA columns: compounds that were irreversible (IRR), e.g. active in carry-over and wash-out (WO) were designated gametocyte-active; compounds that lost activity in wash-out format were designated gamete-active

Independent stocks of 26 of the most potent commercially available hit compounds from both gametocyte and asexual screens, were studied in detail in seven profiling assays (Fig. 2a and Supplementary Data 3) to assess whether they targeted asexual parasite stages, mature gametocytes, male or female gametocytes, male or female gametes or, alternatively, interfered with early mosquito stage development in the rodent malaria parasite *Plasmodium berghei* (or any combination of above). Combining data gave several different activity profiles: gamete-targeted (7 compounds that showed a male-specific gamete-targeted profile), transmission-specific (4 compounds male gametocyte-specific and irreversible), dual asexual-gametocyte targeted (6 compounds, all active against asexual, male and female gametocytes, and were irreversible) and asexual-specific (9 compounds) (Fig. 2b).

The gamete-targeted compounds all specifically inhibited exflagellation (male gamete formation). Moreover, the six N-4HCS compounds comprising most of this category also inhibited *P. berghei* ookinete development (consistent with having prevented exflagellation). The remaining gamete-targeted compound, DDD01062645, was inactive in the ookinete assay possibly indicating specificity for *P. falciparum*. Most of the transmission-specific compounds targeted the male gametocyte specifically, the exception being DDD01254473 that was more potent against female gametocytes than males. It showed reproducible submicromolar activity in the GC-ATP and mitotracker assays, suggesting that DDD01254473 may inhibit a fundamental metabolic pathway more essential to female gametocyte viability than males. Additionally, DDD01255968 also affected male gamete formation, suggesting it may either show polypharmacology or target a biological process common to both stages but absent in asexuals. Five compounds identified in the Pf DGFA primary screen were found to have weak micromolar asexual activity (thus did not meet hit potency criteria in the asexual screen) and so were re-classified as dual asexual/transmission active compounds. Similarly, asexual-specific DDD01027599 showed activity against gametocytes and was also reclassified. Interestingly all but one (DDD01246628) dual asexual/transmission active compounds affected both male and female gametocytes in the Pf DGFA. Coupled to their asexual activity, it is therefore likely that they target biological pathways fundamental for survival in the human host. Supporting this hypothesis, five of the six compounds in the category were also active in one or both of the gametocyte viability assays.

We investigated whether hit compounds could inhibit liver stage invasion and growth in an established *P. berghei* luciferase sporozoite HepG2 invasion assay^24^. Only one compound, DDD01243506, showed submicromolar activity against *P. berghei* liver stage invasion at a similar level to its activity against asexuals (Pb liver IC_50_ = 0.52 µM; HepG2 TOX_50_ ≥ 50 µM; Recombinant luciferase IC_50_ ≥ 50 µM; Pf asexual IC_50_ = 0.66 µM, Supplementary Data 3). Indeed, no Pf DGFA-active compounds showed specific activity against liver stages, presumably either due to fundamental differences in cell biology between different parasite stages or species-specific differences between *P. falciparum* and *P. berghei*.

**Fig. 3:**
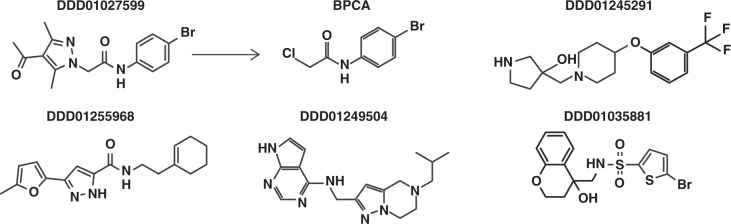
Chemical structure of GHCDL compounds selected for further study. Five compounds showing a range of transmission-blocking activity at the profiling stage were selected for detailed study. DDD01027599 (found to actually be BPCA—see Supplementary Fig. 1-2) and DDD01245291 are active against gametocytes and asexuals; DDD01255968 and DDD01249504 are active specifically against male gametocytes; and DDD01035881 specifically targets male gamete formation

To fully evaluate the power of the screening pipeline and to understand how the profiling categories could translate into transmission-blocking therapeutics, five compounds were selected for further study based upon potency and profile (Fig. 3): DDD01027599 (DDD599/BPCA) and DDD01245291 (DDD291) representing asexual and Pf DGFA dual-stage actives, respectively; DDD01249504 (DDD504) and DDD01255968 (DDD968) representing male-specific and gametocyte-specific actives, respectively; and finally, DDD01035881 (DDD881), one of the N-4HCS cluster representing male gamete-specific actives. In summary, our screening efforts identified 17 confirmed transmission blocking and 9 asexual targeting leads for further development.

### Phenotypic impact of compounds on male gametogenesis

**Fig. 4:**
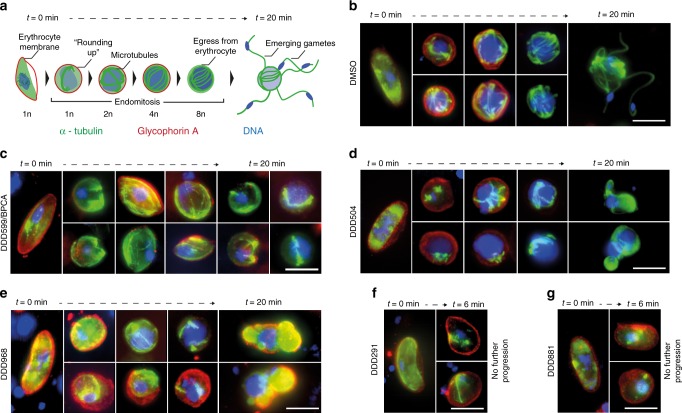
Monitoring the effect of compound treatment on male gamete formation by IFA. Gametocytes were treated for 24 h with DMSO or 10 µM test compound and then gamete formation triggered by the addition of ookinete medium and reduction to room temperature. Aliquots were sampled at different time points and immediately fixed before being stained directly for α-tubulin (green), glycophorin A (red) and DNA via DAPI labelling (blue). Images show representative non-induced cells (0 min), representative cells during gamete formation progression (2–10 min), and representative cells showing the most advanced phenotype at 20 min. **a** Upon activation, male gametocytes initially round up before undergoing three endomitotic genome replications, egressing from the surrounding erythrocyte membrane and releasing up to eight flagellated gametes. **b** Images from left to right show DMSO-treated cells undergoing normal male gamete formation over 20 min. Male gametocytes initially have an elongated morphology with a faint nucleus, relatively homogenous tubulin distribution and are surrounded by an erythrocyte membrane. Upon induction of gametogenesis, cells round up and begin three rounds of endomitosis, with ordered microtubule spindle fibres and increasingly intense DNA staining. Towards the end of gametogenesis, the surrounding erythrocyte membrane is shed and up to eight microgametes emerge from the cell possessing a microtubule-rich flagellum and one replicated genome. **c** DDD599/BPCA-treated cells; **d** DDD504-treated cells; **e** DDD968-treated cells; **f** DDD291-treated cells; **g** DDD881-treated cells. Bars = 5 µm

**Fig. 5:**
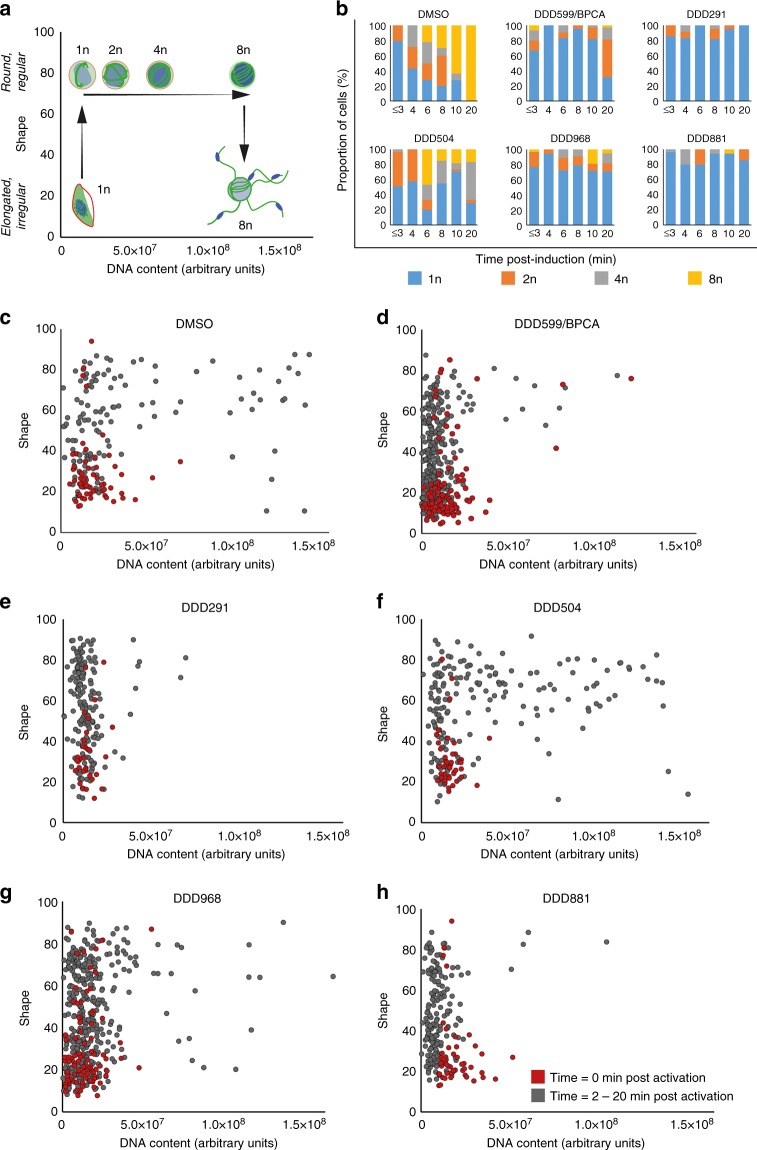
Computer-aided quantification of male gamete formation from IFA images. Individual cells were extracted from IFA images stained for α-tubulin, glycophorin A and DNA from samples fixed between 0 and 20 min post induction of gamete formation (*n* = 195–668 cells per compound treatment). Cell shape (elongated/irregular vs. round) and haploid chromosome number (1nto 8n) were estimated from α-tubulin and DAPI staining (DNA content) respectively (arbitrary units). **a** Schematic diagram illustrating how cell shape and DNA content parameters vary over the course of male gamete formation. Initially gametocytes are elongated with 1n haploid chromosome number. Upon induction, they round up then undergo three rounds of DNA replication whilst maintaining a round shape. Finally, male gamete release (exflagellation) changes the shape of the cell whilst maintaining a high DNA content. **b** Genome copy number during gamete formation was estimated by benchmarking mean DAPI intensity with respect to the non-activated gametocytes at *t* = 0 min (1n) and exflagellating cells at *t* = 10–20 min (8n). DNA replication was compared between the DMSO control and five test compounds. **c**–**h** Graphs plotting the cell shape and DAPI intensity data for each individual cell analysed. Red dots show the initial distribution of cells before induction (*t* = 0 min). Grey dots show induced cells (*t* = 2–20 min)

In vitro evolution followed by whole-genome analysis is the primary approach currently used for understanding how compounds act in malaria parasites^25^. As it is not currently feasible to generate resistant mutants to compounds that are only active in the gametocyte stage (resistance generation requires extensive rounds of replication in the presence of compound), phenotypic immunofluorescence assays were performed to evaluate compound activity on male gamete formation. Gametocytes treated with 10 µM of each compound were sampled at 0, 2, 3, 4, 6, 8, 10, and 20 min post induction of gamete formation before imaging (Fig. 4). Individual male cells were then extracted from the images and analysed for shape and haploid chromosome number/DNA content to quantitatively measure the progression of DNA replication (*n* = 198–668 cells) (Fig. 5a).

Each of the five compounds arrested male gametogenesis but showed distinct morphological failures suggesting distinct modes of action (Fig. 4b–g). DDD599/BPCA-treated gametocytes rounded up after induction but most cells did not replicate their DNA and none exflagellated (Figs. 4c and 5d). Cells frequently showed irregular and distorted tubulin fibres that did not associate with the generally small condensed nucleus. At the last observed time point (20 min post induction), a proportion of cells had commenced DNA replication, possessing microtubule fibres resembling the initial round of endomitosis of the DMSO control and an increase in DNA content (Fig. 5b). Most DDD291-treated male gametocytes also arrested after rounding up (Figs. 4f and 5e). Microtubule staining showed some cells formed spindle fibres however these appeared disordered in the absence of DNA replication (Fig. 4f). Contrastingly, the first round of DNA replication of DDD504-treated male gametocytes appeared with similar kinetics to the DMSO control (Fig. 5b). However, the second and third divisions were delayed with a smaller proportion of cells reaching 8n. Interestingly, microtubule staining appeared to show that during DNA replication, the spindle poles do not separate and rotate into opposition, but cluster at one location rather than being evenly spaced out around the cell periphery. The few cells that do attempt exflagellation did not have properly segregated genomes and no organised flagellar structure (Fig. 4d). Most DDD968-treated male gametocytes did not replicate their DNA and remained with 1n genome copies (Fig. 5b). However, some cells successfully progressed to 8n with disordered appearing spindle fibres and resulted in an irregular-shaped cell possessing a large DNA-rich mass and intense large foci of tubulin-rich cytoplasm that distorted the overlying erythrocyte membrane (Figs. 4e and 5b, g). Finally, the gamete-targeted DDD881, like DDD291, permitted induced gametocytes to round up but only a few cells replicated their DNA and none exflagellated (Fig. 5b, h). In activated cells, the usually diffuse DNA of the gametocyte frequently appeared somewhat condensed with tubulin-rich accumulations at various points surrounding it (Fig. 4g). Although phenotypes do not define a precise mode of action, the distinct nature of each suggests very different cellular targets during male gametogenesis.

### Preliminary chemical profiling, SAR and DMPK analysis

**Fig. 6:**
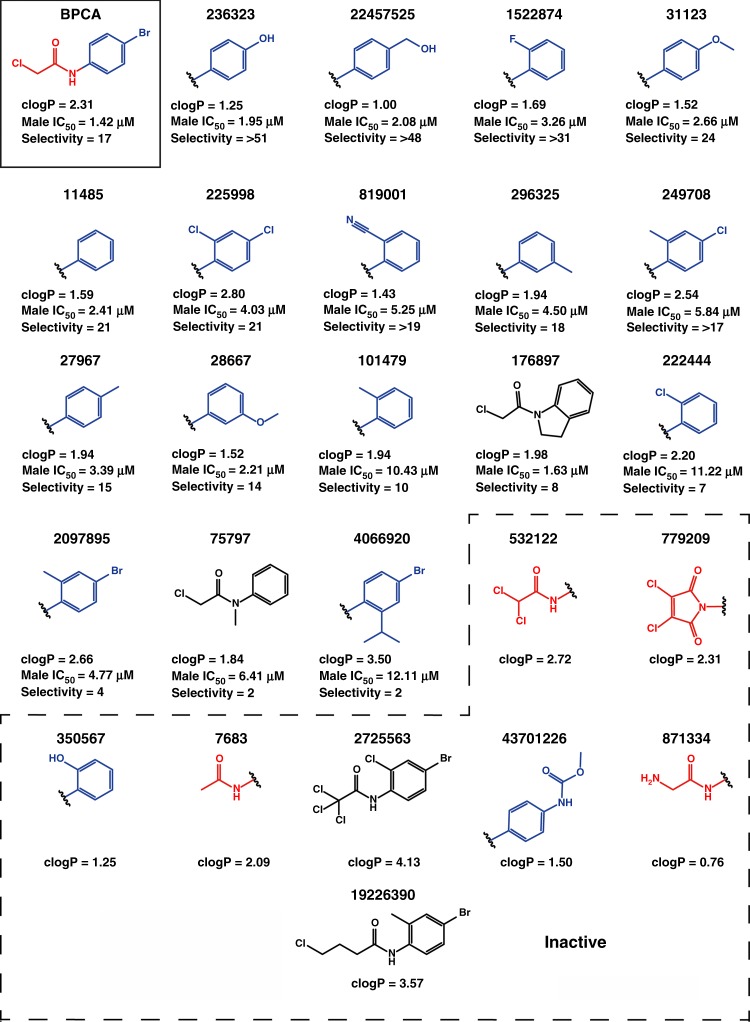
Structure–activity relationship between BPCA/DDD599 and analogues. Activity in the Pf DGFA male readout and parasite selectivity compared to Tox_50_ in HepG2 cells shown. Red and blue groups indicate modifications to respective parts of the parent molecule. Black molecules are modified in both groups. Analogue IDs correspond to PubChem ID (https://pubchem.ncbi.nlm.nih.gov/)

**Fig. 7:**
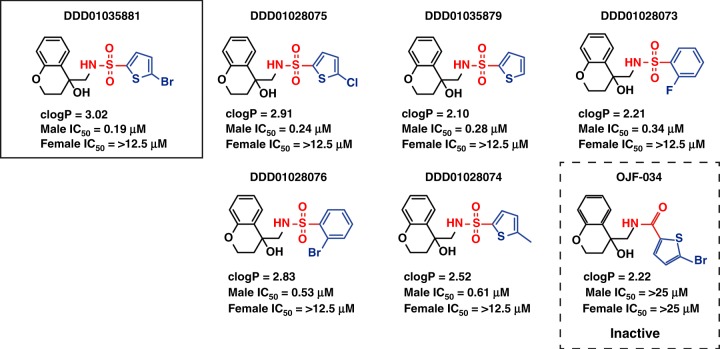
Pf DGFA structure activity relationship between DDD01035881 and analogues. Red and blue groups indicate modifications to respective parts of the parent molecule. Analogue IDs correspond to molecules within the GHCDL with the exception of OJF-034 which was synthetised in-house

Having explored their effects on cell development, each compound was resynthesized either ‘in house’ or re-sourced commercially and their activity re-confirmed in the Pf DGFA (Supplementary Methods and Supplementary Fig. 1-4). Surprisingly freshly resynthesized DDD0504, DDD968 and DDD599 were all inactive in the Pf DGFA. DDD504 and DDD968 were hypothesised to be prone to oxidation and potency of both could be restored by aging the molecules (Supplementary Fig. 5). By ^1^H-NMR, aged compounds appeared to be a mixture of different products and so the identity of the active metabolite(s) could not be resolved. However, preliminary analysis of DDD504 suggests that the most likely position for its oxidation is the 4-unsubstituted position in the pyrazole ring, as seen by the loss of the signal for that proton in the ^1^H-NMR spectra upon aging (Supplementary Fig. 5). Consequently, study of both DDD504 and DDD968 was paused pending future identification of active component(s). The GHCDL-derived stock of DDD599 gave different LCMS and NMR spectra than resynthesized DDD599 and was positively identified as N-(4-bromophenyl)-2-chloroacetamide (BPCA) (Supplementary Fig. 1-2). When adjusted for correct molecular weight, BPCA was found to be almost identically active to GHCDL DDD599 (Asexual IC_50_ = 0.80  vs. 0.80 µM; Pf DGFA male IC_50_ = 1.46  vs. 1.38 µM; Pf DGFA female IC_50_ = 10.98  vs. 3.43 µM, respectively). We hypothesise that BPCA is a by-product of synthesis. Unsurprisingly, BPCA was reactive with nucleophiles (evidenced by being trapped by glutathione, Supplementary Table 1) and analogues lacking the reactive chlorine were inactive (Fig. 6). However, a broad range of activities was found amongst analogues containing the carbon potentially susceptible to nucleophilic attack with anti-parasite activity not correlated with general cytotoxicity. Substituting the bromine atom for a hydroxyl or hydroxymethyl group gave the highest parasite selectivity. No analogues of DDD291 were commercially available to conduct structure activity relationship (SAR) however drug metabolism and pharmacokinetics (DMPK) analysis showed good aqueous solubility (>250 µM), acceptable mouse liver microsome clearance rate (4.2 ml min^−1^ g^−1^ Liver), but poor membrane permeability (5.9 nm s^−1^) in the parallel artificial membrane permeability assay (PAMPA) suggesting oral bioavailability may be low. The sulphonamide R-group of DDD881 was found to be essential for activity and replacement with an amide group resulted in loss of function (Fig. 7 and Supplementary Text). The replacement of the R′-group thiophene ring with benzene was tolerated, and decoration with a single halogen atom enhanced activity (Fig. 7). DDD881 showed good aqueous solubility (>250 µM), with no detectable metabolism in mouse liver microsomes (<0.5 ml^−1^ min^−1^ g Liver) suggesting a suitably promising profile for in vivo efficacy studies.

### Confirmation of transmission-blocking activity in mosquitos

**Fig. 8:**
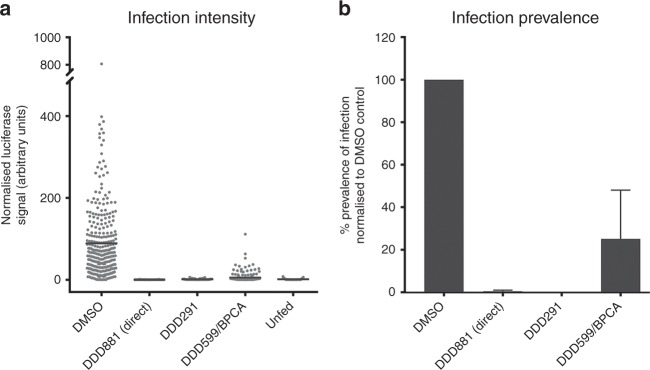
Transmission-blocking efficacy of leads in *P. falciparum* SMFA. The predictive power of the Pf DGFA to identify transmission-blocking compounds was assessed by treating GFP/luciferase-expressing *P. falciparum* gametocytes for 24 h with DMSO, DDD291 or DDD599/BPCA and then feeding them to *A. stephensi* mosquitoes in indirect mode in which test compound is absent from the blood meal. As DDD881 targets gamete formation rather than gametocytes, membrane feeds with this compound were performed in direct mode, added directly to the blood meal with no prior incubation. **a** Normalised luciferase intensity from individual mosquitoes (*n* = 56–72 per condition per replicate) was compared to DMSO and unfed (uninfected) controls. Data combined from three independent biological replicate feeds. Grey dots indicate individual luciferase readout for individual mosquitoes and black bar shows the mean. **b** Calculated infection prevalence of mosquito feeds normalised to respective DMSO control. Error bars denote the standard error of the mean

Compound inhibition in the Pf DGFA accurately predicts inhibition of parasite transmission to the mosquito in the SMFA^23^. To directly measure the transmission-blocking efficacy of our identified hits, we used an established *P. falciparum* SMFA with a luciferase oocyst readout^26^. DDD599/BPCA and DDD291 were tested at 10 µM in indirect mode in which test compounds were incubated with gametocytes for 24 h before being fed to mosquitoes in the absence of compound and presence of oocysts assessed 8 days later (Fig. 8a and Supplementary Data 4). DDD291 and DDD599/BPCA blocked transmission strongly and gave mean normalised luciferase intensities similar to uninfected, unfed mosquitoes (1.37 and 5.27 arbitrary units, respectively; unfed = 1.12; DMSO-treated control = 88.77). Furthermore, the same pattern was observed with prevalence of infection (Fig. 8b), with no infected mosquitoes observed following DDD291 treatment and BPCA treatment reduced infection prevalence to 25.12% of the DMSO control. As DDD881 was found to exclusively target male gamete formation in the Pf DGFA and potency was lost if washed out, it was evaluated in the Pf SMFA at 10 µM in direct mode and consequently was added directly to the blood meal in the membrane feeder without any pre-exposure to gametocytes. Under these conditions DDD881 completely blocked transmission in two out of three replicates and a luciferase reading above uninfected background was only detected in 1 out of 69 mosquitoes in the third replicate (mean normalised luciferase intensity = 0.26; prevalence = 0.49% of DMSO control).

**Fig. 9:**
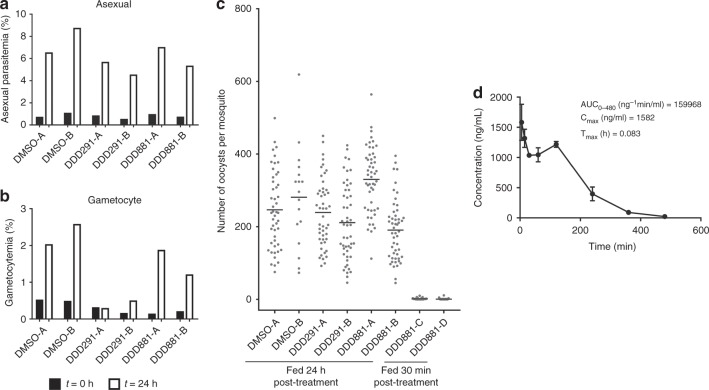
In vivo transmission-blocking efficacy in *P. berghei* direct feeding assays. Eight phenylhydrazine-treated mice were infected with *P. berghei* constitutively expressing GFP. On day 3 of infection, they were assigned into four groups of two mice (DMSO A + B; DDD291 A + B; DDD881 A + B and C + D). Three groups received either 50 mg kg^−1^ DDD291, 50 mg kg^−1^ DDD881 or DMSO + vehicle control by IP injection. Prior to dosing and 24 h later, immediately before feeding *to A. stephensi* mosquitoes, **a** asexual parasitemia and **b** gametocytaemia were recorded. The fourth group received a 50 mg kg^−1^ DDD881 dose on day 4, 30 min prior to mosquito feed. **c** Seven days after feeding, mosquitoes were dissected (*n* = 17–52 per condition) and oocysts recorded by fluorescent microscopy and automated image analysis. Horizontal bars indicate the mean. **d** PK analysis of DDD881. BALB/c mice were dosed with 50 mg kg^−1^ DDD881 (*n* = 3) and plasma concentrations were monitored over time. After an initial distribution phase, DDD881 was eliminated with a half-life of ~90 min. Error bars denote the standard deviation

DDD291 and DDD881 were then evaluated for in vivo transmission-blocking activity in a mouse model of transmission. *P. berghei*-infected mice (*n* = 2 per compound) were given a single intraperitoneal (IP) dose of 50 mg kg^−1^ of DDD291 or DDD0881 on day 3 of infection, 24 h prior to mosquito feed. DDD881 was also administered similarly on day 4 of infection only 30 min prior to mosquito feed in a separate cohort of mice (Supplementary Data 5). Twenty-four-hour exposure to DDD291 did not affect asexual parasitaemia but suppressed gametocytaemia ~6-fold in both mice compared to vehicle-only controls (Fig. 9a, b), however due to the very high baseline gametocytaemia saturating mosquitoes (vehicle control groups had mean oocyst intensities of 246.3 and 281.0 oocysts per mosquito), it was not possible to measure a reduction in transmission at the oocyst level (Fig. 9c). DDD881 specifically targets male gametes therefore it was unsurprising that asexual parasitaemia and gametocytaemia were not impacted by treatment. If administered 24 h before mosquito feeding, DDD881 showed no transmission blocking efficacy at the oocyst level. However, if administered 30 min before the feed, a 99.6% blockade on oocyst intensity was observed. Supporting these observations, pharmacokinetic analysis of naïve mice dosed similarly with DDD881 showed that after an initial distribution phase, plasma concentrations remained stable at ~1000 ng ml^−1^ up to 120 min after dosing before being eliminated, with an approximate half-life of 90 min (Fig. 9d). Combined, these data suggest that, with improved stability or slow release, the N-4HCS scaffold could provide a potent starting point for future transmission-blocking antimalarials.

## Discussion

Using transmission as the first screening filter yielded novel chemical scaffolds that were not identified in the parallel asexual screen and showed inhibition in mosquito transmission assays. This suggests that within the ~7 million compounds already screened by the research community for asexual activity and discarded, there are transmission-blocking molecules still waiting to be discovered. Male gametocytes are much more sensitive to antimalarial compounds than females^23,27^. Likely this is due to the rapid and complex nature of male gamete formation which provides more opportunities for intervention. This is further evidenced by the five distinct inhibition phenotypes of the five compounds studied here (Fig. 4), and also highlights the utility of phenotypic immunofluorescence to shed light on drug targets for which resistance selection is impractical. DDD599/BPCA is a small reactive molecule with poor parasite selectivity. Interestingly however, varying the bromophenyl group greatly affected both activity and parasite selectivity. DDD599/BPCA and analogues therefore may make promising tool molecules for covalent ligand discovery of essential proteins in the parasite genome^28^. DDD504 and DDD968 showed gametocyte-specific activity. Conceptually this class of sexual stage specific antimalarial would be partnered with a schizonticidal combination therapy to both cure malarial symptoms and prevent onward transmission to mosquitoes, effectively protecting both infected persons and the much larger population at risk of infection^11^. Both compounds showed striking phenotypes against male gamete formation (Fig. 4d) and work is now ongoing to identify the active component(s) within these unstable compounds to determine their suitability for further progression. In vitro, DDD291 showed low micromolar activity against asexuals, and both male and female gametocytes (Supplementary Data 3). Transmission was almost totally arrested by DDD291 in the Pf SMFA, however in vivo its effects were less pronounced. Likely the weak in vivo activity is linked to poor membrane solubility of this molecule which could be improved through SAR optimisation.

Of all hits, the N-4HCS scaffold shows the greatest promise as a new chemotype for transmission-specific antimalarial drug development. It is fast-acting, non-toxic and amenable to further medicinal chemistry. Its precise mode of action is unknown; however, it specifically targets early male gamete formation and arrests development before DNA replication. Several other molecules targeting male gamete formation are under development as potential therapeutics^29–31^. Clinical patients treated with current artemisinin-combination therapies (ACTs) even supplemented with a low dose of the only licenced transmission-blocking therapeutic, primaquine, are still infectious to mosquitoes for several days after treatment^32,33^. To immediately halt and maintain a block in transmission, administration of a DDD881-like molecule either with a longer half-life, reformulated for slow release, or administered over multiple doses, could close this transmission window between treatment and complete cure during which drug resistance is being selected for and could escape. Given the low toxicity to human cells and coupled to future improvements in bioavailability/longevity, N-4HCS could be used as a chemical vaccine in mass drug administration campaigns. We envision that in targeted malaria endemic areas, the entire population could be treated with intermittent curative doses of a conventional schizonticidal therapy during the dry season to disrupt the residual parasite reservoir in humans and then use maintenance dose(s) of a DDD881-like molecule to protect the mosquito population from being re-infected as the rainy season begins to suppress new incidences of malarial infection. Based on these findings, we believe further investigation of similar transmission-specific blocking antimalarials is clearly warranted.

## Methods

### Provision of the GHCDL and screening conditions

The Global Health Chemical Diversity Library (GHCDL) was compiled by Dundee University Drug Discovery Unit (DDU) comprising 68,689 compounds with lead-like properties enriched for chemotypes not known to have been previously screened against malaria parasites. The library was provided by DDU dissolved in 100% dimethylsulfoxide (DMSO) in screening-ready 384-well plates (781091, Greiner). Plates arrived frozen and were stored at −20 °C and only thawed immediately prior to use. Chemical structures for the GHCDL can be accessed via the ChEMBL Database^34^. The library was screened in the Pf DGFA with a single replicate at 2 µM with a 48 h pre-incubation of test compounds with stage V gametocytes prior to triggering the gamete formation readout. The initial hit rate was low and so a generous cut-off of >30% inhibition was used yielding 251 hits. Upon re-test in dose response, 24 compounds showed an IC_50_ < 10 µM. The GHCDL was also screened against asexual parasites with a single replicate at 2 µM over 72 h yielding 412 hits with a cut-off of >50% inhibition. Upon retest in dose response, 416 showed an IC_50_ < 10 µM. Compounds were clustered using the OSIRIS DataWarrior using the FragFP algorithm with a 0.8 cut-off.

### Culture of *P. falciparum* asexual and sexual blood stages

*P. falciparum* NF54 strain (originally isolated from an imported malaria case in the Netherlands in the 1980s; BEI Resources, cat. no. MRA-1000) asexual and sexual stages culture was performed as previously described^35^. Gametocyte cultures were initiated at 1% asexual ring stage parasitaemia and 4% haematocrit in 40 ml final volume. To induce gametocyte production, complete culture medium (CM) (RPMI 1640 supplemented with 25 mM HEPES, 50 µg ml^−1^ hypoxanthine, 2 g l^−1^ NaHCO_3_ and 10% pooled human male type A+ serum) was replaced daily for 14 days without fresh erythrocyte addition. Human serum was obtained from Interstate Blood Bank, A^+^ serotype; no aspirin 2 h before drawing and no antimalarials 2 weeks before drawing. On day 14 after culture induction, gametocyte production was assessed by thin smear and Giemsa staining, and exflagellation accurately counted in a haemocytometer^35^. Gametocyte cultures with >0.2% exflagellation (compared to total erythrocyte cell number) were deemed acceptable for use in the Pf DGFA. Note, all human biological samples were sourced ethically and their research use was in accord with the terms of the informed consents under an IRB/EC approved protocol.

### *P. falciparum* dual gamete formation assay (Pf DGFA)

In screening mode, the assay was performed as described in Delves et al.^35^ with a mean Z′ factor of 0.56 ± 0.14 (SD) (male) and 0.71 ± 0.17 (SD) (female). Pooled mature gametocyte cultures with the capacity to yield >0.2% exflagellation (of total cells) were diluted in CM to 14 million cells per ml and so that 700,000 cells were dispensed in 50 µl into each well of the warmed 384-well screening plates. Parasites were incubated in the plates with compounds for 48 h in a humidified chamber under 92%N_2_/5% CO_2_/3% O_2_ gas mix before gamete formation was triggered by addition of 10 µl per well ookinete medium (RPMI 1640 supplemented with 25 mM HEPES, 50 µg ml^−1^ hypoxanthine, 2 g l^−1^ NaHCO_3_, 100 µM xanthurenic acid) containing 0.5 µg ml^−1^ anti-Pfs25 clone 4B7 (BEI Resources, cat. no. MRA-315) conjugated to the fluorophore Cy3 (GE Healthcare), and also by cooling the plate at 4 °C for 4 min and then a further 5 min at 28 °C. Exflagellation was recorded immediately by brightfield microscopy using a Nikon Ti-E widefield microscope at ×4 objective, ×1.5 zoom, recording a 10-frame video time-lapse over 2 s for each well. Afterwards, plates were incubated in the dark at 28 °C for a further 24 h to allow female gametes to maximally express Pfs25 on their surface. Female gamete formation was recorded by single-image fluorescence microscopy using the same microscope and magnification as above. Exflagellation and female gamete formation were quantified by specific algorithms designed with the open-source ICY Image Analysis software^36^. Data were normalised to the percentage of inhibition of the biological response by using positive (12 µM Gentian Violet, Ctrl2) or negative (0.5% DMSO, Ctrl1) controls following Eq. (1) described below:

1 $$\% {\mathrm {Inhibition}} = 100 - \left( {\left( {\left( {{\mathrm {test}}\,{\mathrm {compound}} - {\mathrm {Ctrl2}}} \right)/\left( {{\mathrm {Ctrl1}} - {\mathrm {Ctrl2}}} \right)} \right) \times 100} \right)$$

Assay performance statistics, such as signal to background ratio, Z′ and robust 3×SD activity cut-off were calculated using templates in ActivityBase XE (IDBS, Guilford, Surrey, UK). Hit population analysis and visualisation were conducted using Spotfire DecisionSite (Spotfire, Inc., Somerville, Massachusetts). The pIC_50_ (−log IC_50_) values were obtained using the ActivityBase XE nonlinear regression function in the full curve analysis bundle. In screening mode, the Pf DGFA achieved a mean Z′ factor of 0.56 ± 0.14 (SD) (male) and 0.71 ± 0.17 (SD) (female).

In profiling mode, the Pf DGFA was performed in 96-well plates^23^ in wash-out and add-in formats to aid manual manipulation steps. In the wash-out format, gametocytes were incubated for 24 h with test compounds in round-bottomed 96 well plates and then culture medium replaced three times over 6 h, to remove as much of the compound as possible from the well. Gametocytes were then rapidly transferred to a flat bottomed 96-well plate containing ookinete medium and anti-Pfs25 to trigger gametogenesis and then imaged and quantified as above (in the absence of test compound), hence gametogenesis was assayed in the absence of compound. In add-in format, gametocytes were added directly to flat-bottomed 96-well plates containing test compounds, ookinete medium and anti-Pfs25. Gametogenesis was triggered simultaneously with compound treatment hence only the process of gametogenesis was exposed to compound.

### Whole-cell time extended asexual growth assay

Asexual activity was assayed via *Plasmodium* lactate dehydrogenase activity (pLDH), a surrogate of asexual parasite growth^20^. Cultures of 3D7 strain (BEI Resources, cat. no. MRA-102) asexual blood stages at 0.25% parasitaemia and 2% haematocrit, in RPMI-1640 supplemented with 5% Albumax and 150 µM hypoxanthine, were dispensed into 384-well plates (25 µl per well) containing the test compounds. 2 µM artesunate and DMSO were used as the positive and negative controls, respectively. Parasites were incubated for 72 h at 37 °C, under 5% CO_2_/5% O_2_/90% N_2_ and then frozen at −70 °C. Plates were warmed at RT and 70 µl per well of reaction solution (100 mM sodium l-lactate, 100 mM 3-acetylpyridine adenine dinucleotide (APAD), 125 µM NitroBlue tetrazolium (NBT), 200 µl ml^−1^ diaphorase, 0.5% tween 20, 100 mM Tris-HCl pH 8) were added. After plate mixing, NBT reduction was measured by absorbance at 650 nm using Envision (Perkin Elmer).

### Gametocytocidal ATP-based bioluminescence assay (GC-ATP)

The metabolic viability of late stage gametocytes was determined using a gametocyte ATP bioluminescence assay^16^. Stage IV–V 3D7 gametocyte cultures were double-purified by density gradient sedimentation over NycoPrep® followed by magnetic isolation. Then, parasites (50 µl per well containing 12,500 gametocytes) were added to compound pre-dispensed 384-well plates (781091, Greiner) and incubated at 37 °C for 48 h under 5% CO_2_/3% O_2_/92% N_2_. BacTiter-Glo® kit (G8231, Promega) was used to determine the ATP levels of viable parasites according to manufacturer’s instructions. After reagent addition (50 µl per well), luminescence of the plates was measured using a microplate reader (HTS counter Victor, Wallac).

### Gametocytocidal MitoTracker-based viability assay

As another independent assessment of the metabolic viability of compound-treated stage V gametocytes, the Saponin-lysis Sexual Stage Assay (SaLSSA) was employed^7^. A volume of 100 nl of test compounds were pre-dispensed in 384-well plates (781091-2B, Greiner) in 12-point dose response (25 µM to 1.41 × 10^−4^ µM), prior to the addition of synchronous NF54 stage V gametocytes (40 µl at 0.70% gametocytaemia). Assay plates were then incubated for 72 h in a low oxygen environment (92% N_2_, 5% CO_2_, 3%O_2_) before a 10 µl MitoTracker Red CMXRos (2.5 µM) and saponin solution (0.13%w/v) was added. Plates were returned to 37 °C for 1 h to allow for complete red blood cell lysis, after which viable gametocytes were detected using an Operetta high-content imaging system. Analysis was performed using the Harmony package software, with potency calculated relative to puromycin (positive control) and DMSO (negative control).

### *P. berghei* ookinete development assay (Pb ODA)

All mouse procedures were performed in accordance with the UK Animals (Scientific Procedures) Act (PPL 70/7185) and approved by the Imperial College Animal Welfare and Ethical Review Body (AWERB). The ability of test compounds to inhibit *P. berghei* early mosquito stage development was tested in the Ookinete Development Assay^37^ with a few exceptions. Anaesthetised 6- to 8-week-old female Tucks Ordinary (TO) mice infected with *P. berghei* strain CTRPpGFP^38^ were rapidly exsanguinated by cardiac puncture and 500 µl of blood diluted in 25 ml of ookinete medium supplemented with 20% foetal bovine serum. Immediately the blood/medium mixture was dispensed by multichannel pipette into 384 well plates containing pre-plated test compounds. 10 µM cycloheximide and DMSO were used as the positive and negative controls, respectively. Plates were stored in the dark in a humidified incubator at 18 °C for 24 h. Ookinete production was imaged by fluorescence microscopy of GFP expression under the ookinete-specific CTRP promoter using a Nikon Ti-E widefield microscope and ×10 objective. Ookinete production was quantified using ICY and percentage inhibition calculated as above with reference to positive and negative controls.

### Determination of compound activity profile

Compounds were classified as asexual-specific if they showed IC_50_s < 12.5 µM in the asexual assay but no activity in any other profiling assay. Compounds were classified as dual asexual-gametocyte targeted if they possessed asexual activity as above but also an IC_50_ < 12.5 µM against male or female gametocytes in the 48 h Pf DGFA carry-over format and maintained potency after washing out in the wash-out format and had no activity in the add-in format. Compounds were classified as transmission-specific if they were inactive up to 20 µM against asexual parasites but showed IC_50_s < 12.5 µM against male or female gametocytes in the 48 h Pf DGFA carry-over format and maintained potency after washing out in the wash-out format and had no activity in the add-in format (with the exception of DDD968 which was also active in the add-in format). Compounds were classified as gamete-targeted if they were inactive against asexuals up to 20 µM but showed IC_50_s < 12.5 µM against male or female gametocytes in the 48 h Pf DGFA carry-over format and also activity >12.5 µM in the add-in format but became inactive if washed-out.

### *P. falciparum* SMFA

SMFAs were performed commercially by TropiQ Health Sciences (Nijmegen, The Netherlands)^26^. Cultures of mature stage V gametocytes of *P. falciparum* expressing a GFP-Luciferase fusion protein under the control of the *hsp70* promoter were treated with DMSO or 10 µM of test compound for 24 h (indirect mode). They were then mixed with uninfected erythrocytes and pelleted. The cell pellet was resuspended in human serum to form the blood meal and fed to *Anopheles stephensi* mosquitoes. For direct feeding, gametocytes did not receive pre-exposure to test compounds and test compound was added directly to the blood meal immediately prior to mosquito feeding. Eight days after infection, mosquitoes were individually processed and their lysate assessed for luciferase activity in comparison to uninfected mosquitoes.

### *P. berghei* mosquito feeding assays

Six- to eight-week female T0 mice were treated with phenylhydrazine 3 days prior to intraperitoneal (IP) inoculation with 100 µl of mouse blood infected with the constitutively GFP expressing *P. berghei* 507 strain^39^. On day 3 after infection, thin blood smears were taken from tail blood to quantify parasitaemia and gametocytaemia. Mice were divided randomly into groups of two, received IP injections of 50 mg kg^−1^ of test compounds or vehicle control (33% DMSO in dH_2_O) and fed to *Anopheles stephensi* mosquitoes 24 h later. The exception was two mice that were dosed with DDD01035881 on day 4 of infection only 30 min prior to mosquito feeding. Twenty-four hours after feeding, unfed mosquitoes were removed and remaining mosquitoes maintained for a further 9 days on sucrose solution. Mosquito midguts were then dissected, imaged and oocysts quantified by semi-automated computer analysis^40^.

### Hep G2 cytotoxicity assay

Actively growing HepG2 human cells (85011430, European Collection of Authenticated Cell Cultures) were detached from the culture surface with trypsin and counted using a Cellometer (Nexcelom). Then, 12 ml of culture per plate required were prepared at a density of 1.0 × 10^5^ cells per ml using Minimum Essential Medium (41090, GIBCO) supplemented with 10% foetal bovine serum (10106–169, GIBCO) and 1% NEAA solution (11140–035, GIBCO). Cells were seeded (25 µl per well) in 384-well plates (781098, Greiner) with pre-stamped compounds and controls (DMSO and Doxorubicin 50 µM) (125 nl per well) using a WellMate dispenser (Thermo Scientific). Cells were incubated at 37 °C and 5% CO_2_ in a humidified incubator for 48 h. After incubation, 5 µl of Resazurin (R7017, Sigma) per well (45 µM final concentration) were added and plates were kept at 37 °C for 3.5–4 h. Plates were then read on Pherastar LS plate reader (BMG) and percentage of inhibition for each test compound was calculated using Eq. (2):

2 $$\% {\mathrm {Inhibition}} = 100 - ( ( ( {\mathrm {TEST}}\,{\mathrm {COMPOUND}} - {\mathrm {BLANK}})/\\ ( {{\mathrm {DMSO}}\,{\mathrm {control}} - {\mathrm {BLANK}}})) \times {100})$$

TOX_50_s were calculated using ActivityBase templates.

### *P. berghei* liver stage assay

Compound inhibition of *P. berghei* sporozoite liver infection was assessed in a high-throughput luciferase assay^24^. 1536-well plates (789173-F, Greiner Bio-One) were pre-seeded with 5 µl HepG2-A16-CD81^EGFP^ cells grown in DMEM (11965–092, Invitrogen) containing 10% FBS (35–011-CV, Corning) and 1× Pen Strep Glutamine (10378–016, Invitrogen). Then, 50 nl of compound in DMSO was dispensed in 12-point serial dilution dose-response format (50 µM to 2.82 × 10^−4^ µM), and incubated overnight*.A. stephensi* mosquitoes infected with *P. berghei*-ANKA-GFP-Luc-SM_CON_ (Pb-Luc)^41^ sporozoites bearing the antifolate-resistance-conferring *Toxoplasma gondii* dihydrofolate reductase-thymidylate synthase selectable marker, pyR2. After dissection, ~1000 sporozoites in 5 µl screening media (DMEM, 11965–092, Invitrogen; 5% FBS, 35–011-CV, Corning; 5x Pen Strep Glutamine, 10378–016, Invitrogen) were added per well and incubated for 48 h (37 °C, 5% CO_2_). Bioluminescence was measured using an EnVision microplate reader (Perkin Elmer), following the addition of 2 µl per well of Bright-Glo™ (Promega). Compound toxicity against HepG2 cells was determined in parallel by measuring cellular ATP levels reported by luciferase activity. Methods were similar to the *P. berghei* liver stage assay, except for the addition of CellTiter-Glo® (Promega) as a detection reagent. DMSO (0.5%) was used as a negative control in both assays, with Atovaquone (5 µM) and Puromycin (10 µM) acting as positive controls in the *P. berghei* and toxicity assays, respectively.

### Recombinant luciferase inhibition assay

A counter-screen to the *P. berghei* liver stage assay was run against recombinant luciferase (*Photinus pyralis*) to measure the intrinsic bioluminescence inhibition from select compounds. Briefly, compounds were plated in 12-point serial dose response (50–2.82 × 10^−4^ µM) in 1536-well opaque-bottom plates (789173-F, Greiner Bio-One). A 20 pM QuantiLum® Recombinant Luciferase (E1701, Promega) containing solution was made in screening media (DMEM, 11965–092, Invitrogen; 5% FBS, 35–011-CV, Corning; 5x Pen Strep Glutamine, 10378–016, Invitrogen), and 8 µl were added to each well. After 1 h incubation at room temperature, 2 µl of Bright-Glo™ (E2610, Promega) were dispensed per well, and bioluminescence was immediately measured using the EnVision microplate reader (PerkinElmer). DMSO (0.5%) and Luciferase Inhibitor-2 (9.8 µM) (119114, MilliporeSigma) served as negative and positive controls, respectively.

### Immunofluorescence staining and modelling of exflagellation

Day 14 gametocyte culture was divided into 4 ml aliquots and treated either with DMSO or 10 µM test compound. After 24 h, 500 µl aliquots of the non-activated gametocytes were fixed in an equal volume of 8% paraformaldehyde (PFA) warmed to 37 °C to generate Time = 0 min (*t* = 0) samples. Gamete formation was induced in the remainder of the cultures by cooling to RT and addition of 500 µl ookinete medium. At *t* = 2, 3, 4, 6, 8, 10, and 20 min 500 µl of culture was removed and immediately fixed in equal volume of 8% PFA at room temperature for 15 min. Fixed cells were diluted in PBS and allowed to settle onto poly-l-lysine-coated glass coverslips overnight. The next day, cells were washed in PBS, permeabilized in 0.1% Triton X-100 and blocked in PBS + 10% FBS. Cells were stained with mouse anti-alpha tubulin (Sigma) clone DM1A (1:500 dilution) and rabbit anti-Glycophorin A (Abcam) clone EPR8200 (1:1000 dilution). Staining was visualised with anti-mouse Alexa448 and anti-rabbit Alexa555 (Thermo-Fisher) both at 1:1000 dilution. Coverslips were mounted onto glass slides with VectaShield mountant (Vector Laboratories) containing 4′,6-diamidino-2-phenylindole (DAPI) to visualise cellular DNA. Cells were imaged at random (*n* = 195–668 cells per test compound) using a Nikon Ti-E widefield microscope using a × 100 objective lens and taken in 0.2 µm slice z-stack images to capture the whole cell. All images were converted to maximum intensity projections in NIS Elements v4.20 and all further image processing performed in ICY. Individual cells were manually selected and automatically extracted from each image using the Active Contours plugin gated to the tubulin staining. Using the ROI Statistics plugin, the following parameters were calculated for each cell and exported to MS Excel: Cell area, Cell roundness, Cell elongation, and Average intensity of blue channel (DNA). In the DMSO control image collection, it was found that Cell roundness and Cell elongation were proportional and excellent discriminators of falciform-shaped non-activated gametocytes and round activated gametocytes. Outlier cells from the curve were all exflagellating (Supplementary Fig. 6). These two parameters were combined as a ratio of roundness:elongation to produce the parameter Cell shape. Cellular DNA content was estimated by multiplying the average pixel intensity of the blue channel (after subtracting the background intensity of an empty area in the same parent image) with the total cell area.

### Generation of active DDD504 and DDD698 by aging

Freshly synthesised powder was dissolved to 30 mM in DMSO and aged for 10 days at room temperature in the light or 10 days at 37 °C in the dark with tube caps opened every 2 days to allow air to circulate. In the light, potency of DDD504 was restored giving an IC_50_ of 381 nM (compared to the untreated compound and compound incubated in the dark at 37 °C that were inactive). Contrastingly, light did not affect DDD986, however restoration of activity appeared to be linked to temperature as prolonged incubation at 37 °C yielded greater activity than when incubated at room temperature (IC_50_ = 5.85  and 15.9 µM, respectively).

### DMPK and compound stability analysis

DMPK analyses were performed by the Dundee University Drug Discovery Unit and BPCA/DDD599 stability analysis were undertaken by chemists at GlaxoSmithKline using in-house, standardised approaches^42^.

### Code availability

All computer codes used to analyse the data in this study are available from the corresponding authors upon reasonable request.

### Data availability

The data that support the findings of this study are available from the corresponding authors upon reasonable request.

## Electronic supplementary material

Supplementary Information

Description of Additional Supplementary Files

Supplementary Data 1

Supplementary Data 2

Supplementary Data 3

Supplementary Data 4

Supplementary Data 5
