# Supplementary material for: A high throughput screen for next-generation leads targeting malaria parasite transmission
Source: Nat Commun. 2018 Sep 18;9:3805. doi: 10.1038/s41467-018-05777-2 (PMC6143625; doi:10.1038/s41467-018-05777-2)
Supplement: Supplementary file 1 — Supplementary Information [file 41467_2018_5777_MOESM1_ESM.pdf]

Supplementary Information for

A high throughput screen for next-generation leads targeting malaria parasite transmission

Michael J. Delves[1]†, Celia Miguel-Blanco[1,2]†, Holly Matthews[1], Irene Molina[2], Andrea Ruecker[1], Sabrina Yahiya[1], Ursula Straschil[1], Matthew Abraham[3], María Luisa León[2], Oliver J. Fischer[4], Ainoa Rueda-Zubiaurre[4], Jochen R. Brandt[4], Álvaro Cortés[2], Anna Barnard[4], Matthew J. Fuchter[4], Félix Calderón[2], Elizabeth A. Winzeler[3], Robert E. Sinden[1], Esperanza Herreros[2], Francisco J. Gamo[2]* and Jake Baum[1]*

Correspondence to: Jake Baum (jake.baum@imperial.ac.uk), Department of Life Sciences, Imperial College London, Exhibition Road, South Kensington, London SW7 2AZ, United Kingdom; Dr Francisco Javier Gamo (francisco-javier.b.gamo@gsk.com) GSK, C. Severo Ochoa 2, Tres Cantos 28760, Spain.

This file includes:

Supplementary Methods

Supplementary Figures 1 to 6

Supplementary Table 1

Supplementary References

SUPPLEMENTARY METHODS

Abbreviations: 1-Ethyl-3-(3-dimethylaminopropyl)carbodiimide (EDC); Hydroxybenzotriazole (HOBt); Dimethylformamide (DMF); Dichloromethane (DCM); Ethyl acetate (AcOEt); Petroleum ether (PE)

Identification of commercial DDD01027599 as N-(4-bromophenyl)-2-chloroacetamide (BPCA)

After promising compound profiling with commercially available DDD01027599 (Life Chemicals, F3316-0293), compound re-synthesis was carried out internally at GlaxoSmithKline (GSK) by the following route:

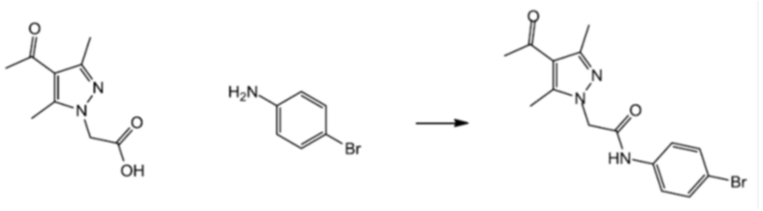

Over a stirred mixture of 2-(4-acetyl-3,5-dimethyl-1H-pyrazol-1-yl)acetic acid (100mg, 0.51mmol), HOBT (0.61mmol), EDC (0.51mmol) and DIEA (0.18ml, 1.09mmol) in DMF (3 mL), 4-bromoaniline (0.51mmol) was added. The reaction mixture was stirred at room temperature for 24h. The mixture was partitioned between ethyl acetate (3 mL) and 1N aqueous NH_4Cl (3mL). The phases were separated and the aqueous one was re-extracted with ethyl acetate. The organic layers were combined and washed with saturated NaHCO3, dried over Na_2SO_4, filtered and evaporated. Crude was purified by chromatography (cyclohexane/AcOEt) affording the desired compound.

The structure of the final synthesised compound was confirmed by NMR and LC/MS analysis. However, unlike commercial DDD01027599, it was inactive against asexuals and male/female gametocytes (IC_{50} >50uM). When the LC/MS and NMR spectra for both compounds were compared, significant differences were observed (Figures S1 + S2).

Synthesis of OJF-034, in house derivative of DDD01035881

5-bromo-2-thiophenecarboxylic acid (104 mg, 0.55 mmol), EDC (71 mg, 0.68 mmol) and HOBt (92 mg, 0.68 mmol) were dissolved in anhydrous DMF (10 mL) under an argon atmosphere and cooled to

0°C. 4-(aminomethyl)chroman-4-ol (80 mg, 0.45 mmol) dissolved in anhydrous DCM (2 mL) was then added followed by trimethylamine. The reaction mixture was then stirred at 0°C for 30 minutes before being warmed to room temperature and stirred overnight. The solution was then diluted with DCM (80 mL) and washed with LiCl (5%, 3 x 100 mL) and brine (100 mL). The organic phase was dried over $MgSO_4$, filtered and the filtrate evaporated to give the crude product as a yellow oil. The product was purified via flash column chromatography on deactivated silica (PE: Et_2O – 1:3) and was isolated as a white solid (81 mg, 0.25 mmol, 55%). ^1H NMR (400 MHz, Methanol-d4) δ 2.03 (ddd, *J* = 14.2, 8.8, 3.9 Hz, 1H), 2.15 (ddd, *J* = 13.9, 6.3, 3.2 Hz, 1H), 3.65 (d, *J* = 14.0 Hz, 1H), 3.86 (d, *J* = 14.0 Hz, 1H), 4.21 – 4.36 (m, 2H), 6.80 (dd, *J* = 8.2, 1.3 Hz, 1H), 6.94 (td, *J* = 7.6, 7.6, 1.2 Hz, 1H), 7.15 – 7.21 (m, 2H), 7.50 – 7.56 (m, 2H). ^{13}C NMR (101 MHz, Methanol-d4) δ 32.85, 63.19, 68.56, 116.37, 117.79, 120.09, 126.21, 126.80, 128.75, 128.85, 130.91, 140.44, 154.49, 162.32. MS: *m/z* (ES) 366 $[M-H]^-$. HRMS, found 365.9771 ($C_{15}H_{13}NO_3SBr$, $[M-H]^-$, requires 365.9756). TLC (hexane:EtOAc - 1:1) R_f: 0.29.

SUPPLEMENTARY FIGURES

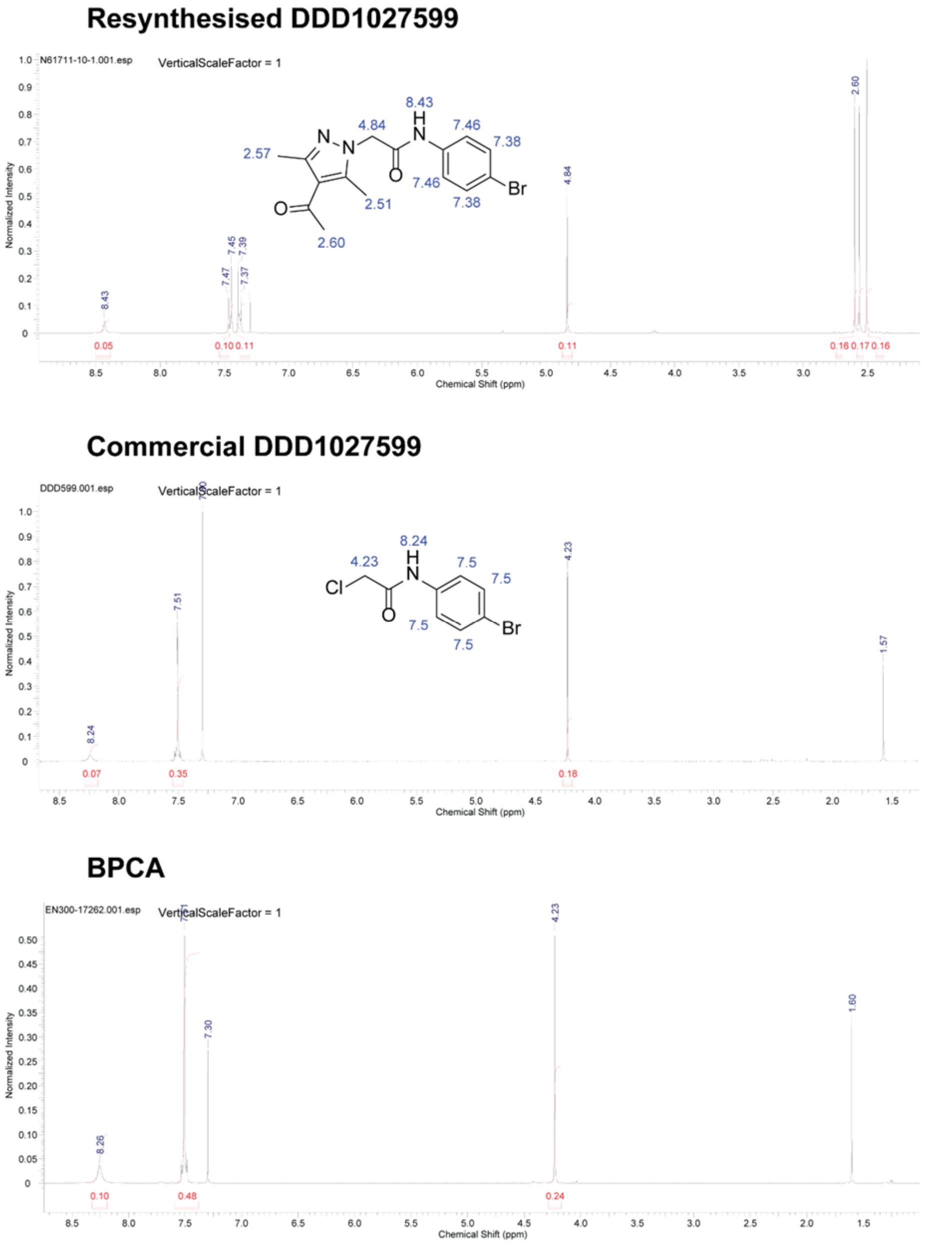

Supplementary Figure 1 I Comparative NMR spectra for internally synthesised DDD01027599, commercial DDD01027599 and N-(4-bromophenyl)-2-chloroacetamide (BPCA).

Commercial DDD01027599 possessed peaks corresponding to the aromatic ring (around 7.5ppm), but peaks corresponding to the methyl groups (around 2.5 ppm) were absent, suggesting that the 3,5 dimethyl 1-H pyrazol moiety was not present in this compound.

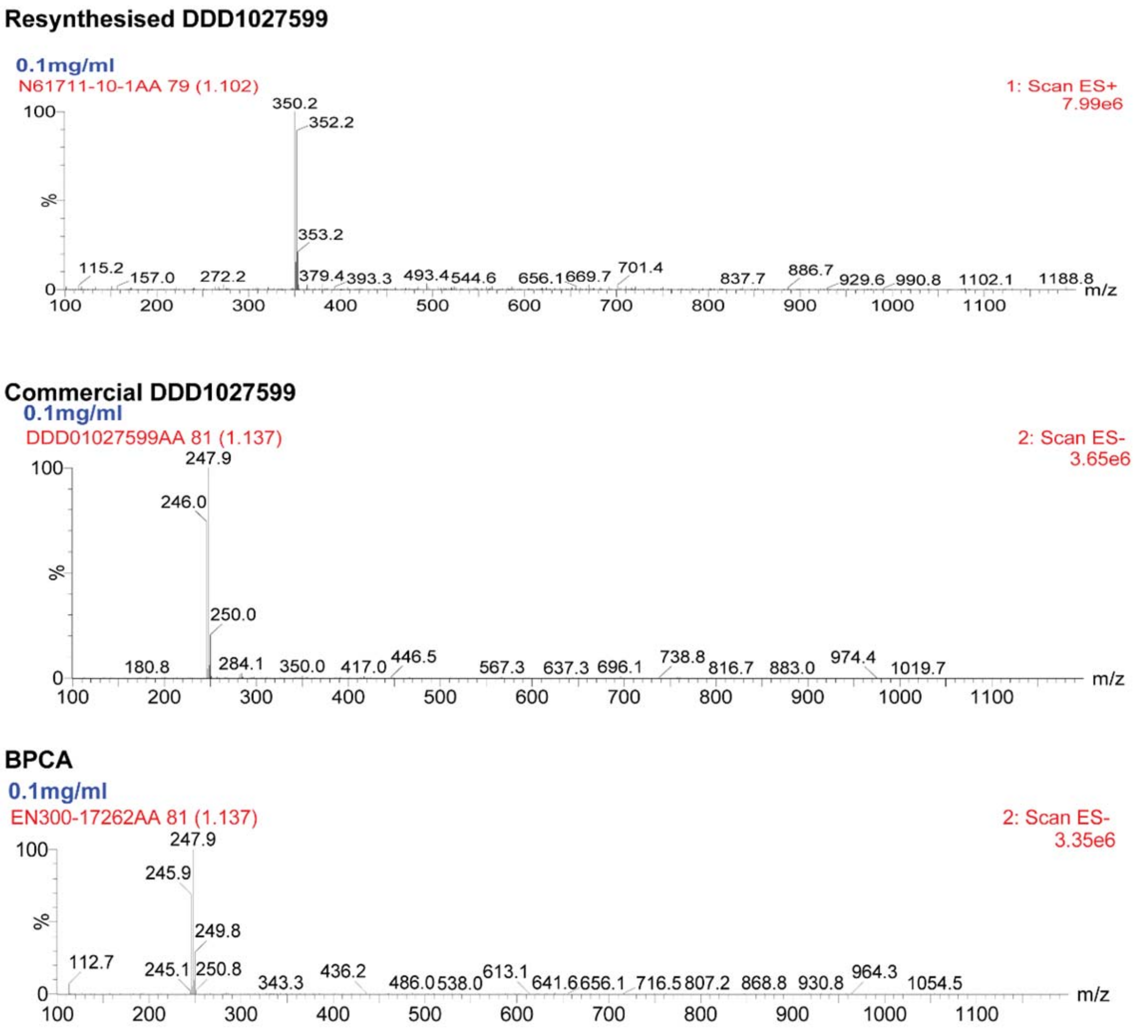

Supplementary Figure 2 I Comparative LCMS spectra for internally synthesised DDD01027599, commercial DDD01027599 and N-(4-bromophenyl)-2-chloroacetamide (BPCA).

LC/MS analysis showed that whilst re-synthesised DDD01027599 had a molecular weight of ~350 (expected to be 350.22), commercial DDD01027599 had a molecular weight of ~248. In addition, high-resolution MS spectrum for DDD01027599 showed the typical pattern for a bromine/chlorine combination. Based on this information the most likely structure proposed for commercial DDD01027599 was N-(4-bromophenyl)-2-chloroacetamide (BPCA) (as shown in Supplementary Fig. 1).

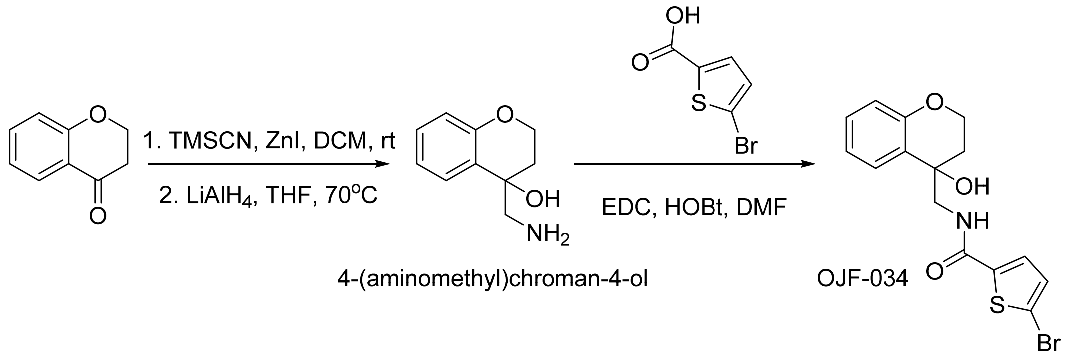

Supplementary Figure 3 I Synthesis of OJF-034.

OJF-034, an analogue of DDD881 possessing an amide rather than sulphonamide linker was synthesised from 4-chromanone a 70% yield over 2 steps according to literature procedures[1,2].

a.

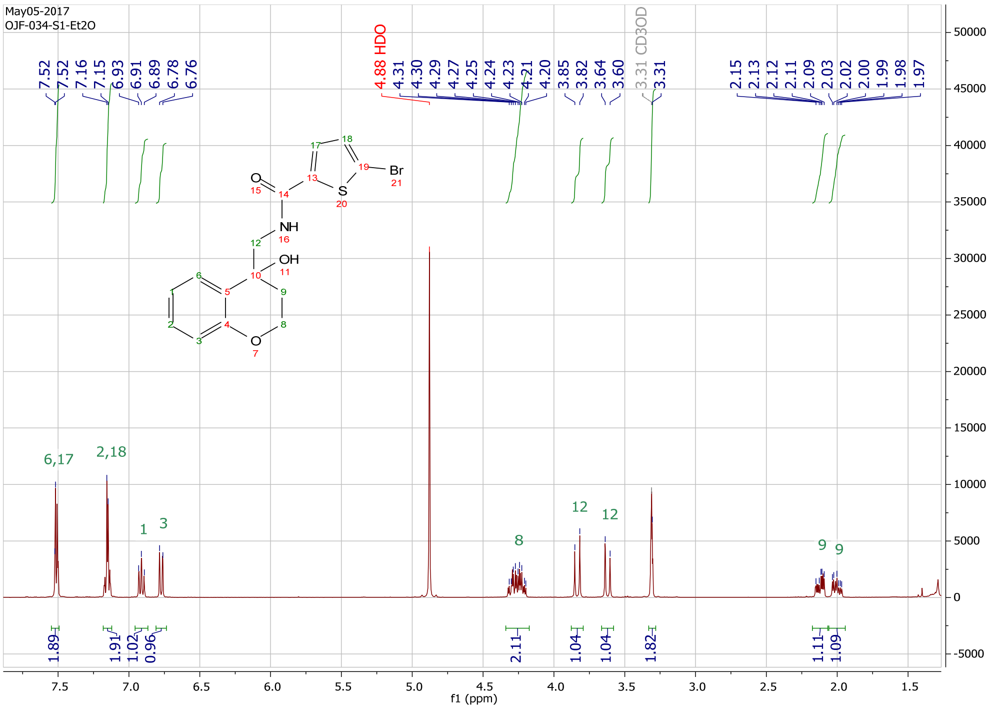

b.

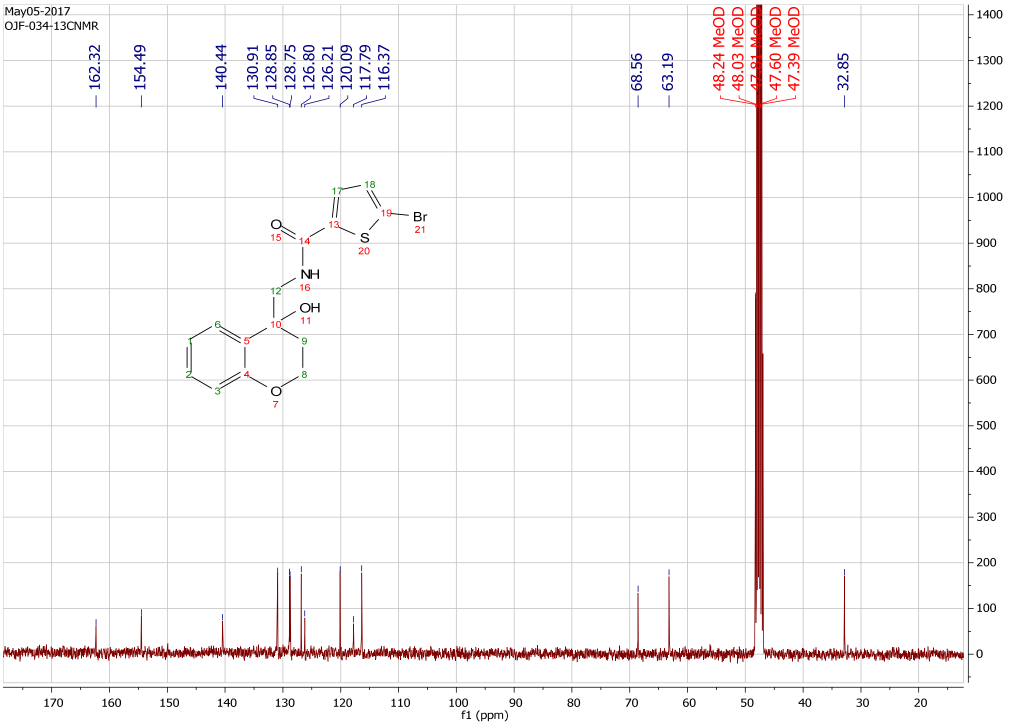

Supplementary Figure 4 I Structural analysis of OJF-034

The structure of OJF-034 was confirmed by: (a) ^{1}H NMR Spectra of OJF-034 and (b) ^{13}C NMR Spectra.

a.

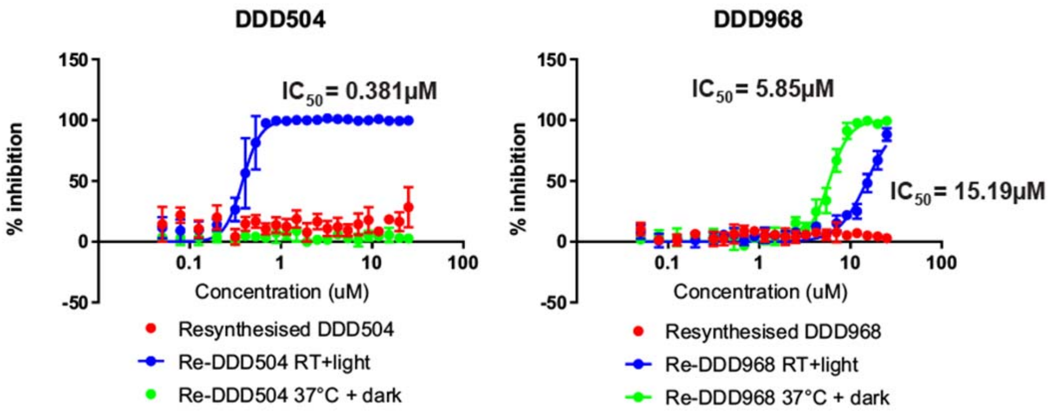
DDD504
IC_{50}= 0.381μM
% inhibition
Concentration (uM)
Resynthesised DDD504
Re-DDD504 RT+light
Re-DDD504 37°C + dark
DDD968
IC_{50}= 5.85μM
IC_{50}= 15.19μM
Resynthesised DDD968
Re-DDD968 RT+light
Re-DDD968 37°C + dark

b.

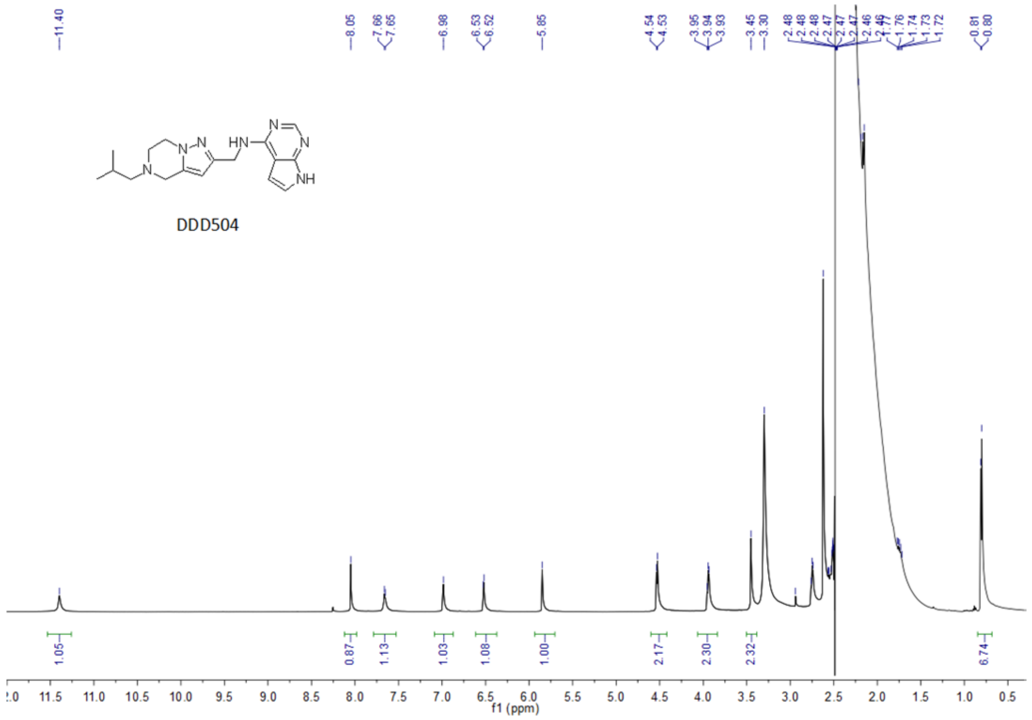
DDD504
f1 (ppm)

c.

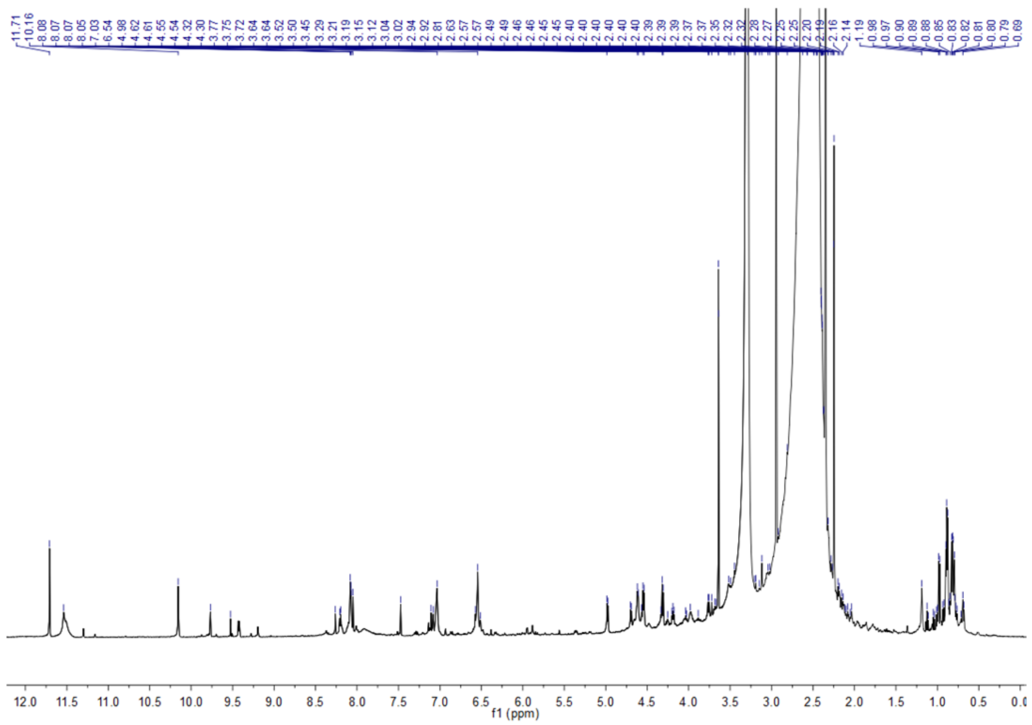

Supplementary Figure 5 I The effect of aging on DDD504 and DDD968.

(**a**) Re-synthesised DDD504 and DDD968 were inactive when re-tested in the Pf DGFA. It was hypothesised that both could be prone to oxidation and/or decomposition and so both molecules were aged to attempt to generate active by-products. Red = resynthesized compound. Blue = resynthesized compound after 10 days at room temperature in the light. Green = resynthesized compound after 10 days at 37°C in the dark. Error bars denote the standard deviation. (**b**) Structure activity relationship between DDD504 and analogues in the Pf DGFA. ^{1}H-NMR spectra of compound DDD504 before and after submitting it to the aging process. Clean spectra of the synthesized compound in DMSO. (**c**) Spectra of the aged compound showing a complex mixture. The loss of the signal at 5.85 ppm could be observed.

a.

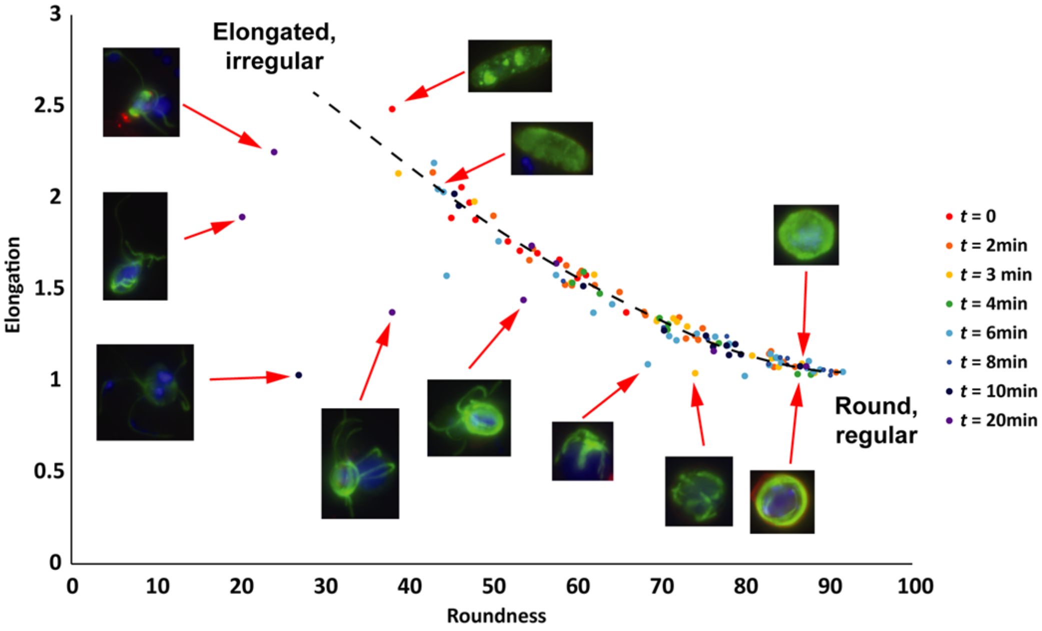

b.

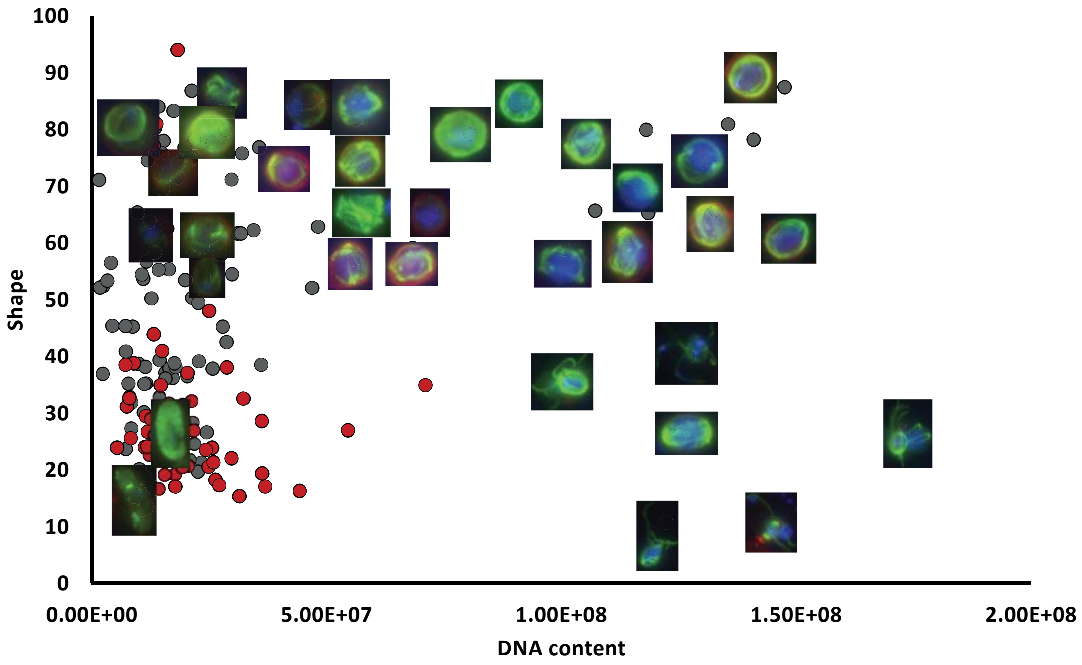

Supplementary Figure 6 I The relationship between cell roundness, elongation and DNA content.

(**a**) With the progression of male gametogenesis, activated cells round up and lose their falciform shape. This transformation in roundness and regularity is initially proportional. However, as gametes begin to emerge, the proportionality is destroyed and exflagellating cells can be easily identified by position on the graph. (**b**) As Figure 4C from the main text with raw images overlaid onto selected data points to illustrate typical phenotypes of cells undergoing male gametogenesis over time. Red dots show the initial distribution of cells before induction (*t* = 0 min) and grey dots show induced cells (*t* = 2-20 min).

SUPPLEMENTARY TABLES

Supplementary Table 1. Stability analysis of DDD599/BPCA

	pH10 Ox	pH10	pH6 Ox	pH6	pH4 Ox	pH4	SGF Ox	SGF	GSH
**Half-life**	92 hours	94 hours	>1000 hours	>1000 hours	>1000 hours	>1000 hours	>1000 hours	607 hours	<1 hours
**t10% degradation / hours**	18 hours	18 hours	>1000 hours	>1000 hours	>1000 hours	>1000 hours	512 hours	118 hours	<1 hours
**% remaining at 24h**	87%	87%	100%	100%	100%	100%	99%	98%	0%

KEY

pH10 Ox = Britton Robinson pH10 buffer + 20M% H2O2
pH10 = Britton Robinson pH10 buffer
pH6 Ox = Britton Robinson pH6 buffer + 20M% H2O2
pH6 = Britton Robinson pH6 buffer
pH4 Ox = Britton Robinson pH4 buffer + 20M% H2O2
pH4 = Britton Robinson pH4
SGF Ox = Simulated Gastric Fluid + 20M% H2O2
SGF = Simulated Gastric Fluid
GSH = Phosphate buffer pH7.4 with 5mM Glutathione

The stability of BPCA was tested in several pH buffers and simulated gastric fluid all with or without an oxidising environment. Additionally, it was also tested in the presence of glutathione. Green text denoted acceptable stability and red text denotes unacceptable stability under test conditions. At pH10 regardless of the presence of H_2O_2, DDD599/BPCA was unfavourably unstable. Similarly, DDD599/BPCA is rapidly cleared in the presence of glutathione with mass spec analysis showing de-chlorination of DDD599/BPCA and covalent attachment to glutathione.

SUPPLEMENTARY REFERENCES

1 Glennon, R. A. & Liebowitz, S. M. Serotonin receptor affinity of cathinone and related analogues. *J Med Chem* **25**, 393-397 (1982).

2 Ornstein, P. L. *et al.* 2-substituted (2SR)-2-amino-2-((1SR,2SR)-2-carboxycycloprop-1-yl)glycines as potent and selective antagonists of group II metabotropic glutamate receptors. 2. Effects of aromatic substitution, pharmacological characterization, and bioavailability. *J Med Chem* **41**, 358-378, doi:10.1021/jm970498o (1998).
