# Supplementary material for: A high throughput screen for next-generation leads targeting malaria parasite transmission
Source: Nat Commun. 2018 Sep 18;9:3805. doi: 10.1038/s41467-018-05777-2 (PMC6143625; doi:10.1038/s41467-018-05777-2)
Supplement: Supplementary file 2 — Description of Additional Supplementary Files [file 41467_2018_5777_MOESM2_ESM.docx]

**Description of Additional Supplementary Files**

File Name: Supplementary Data 1
Description: **Confirmed hits in the GHCDL Pf DGFA screen.** The GHCDL was screened in the Pf DGFA in a single replicate at 2 µM with a 48 h gametocyte incubation prior to triggering gametogenesis. Compounds giving >30% inhibition in either the male or female readout were retested in dose response and those with an IC_50_ <10 µM were determined.

File Name: Supplementary Data 2
Description: **Confirmed hits in the GHCDL Pf Asexual screen**. The GHCDL was screened for inhibition of *P. falciparum* asexual growth in a single replicate at 2 µM with a 72 h compound incubation period. Compounds giving >30% inhibition retested in dose response and those with an IC_50_ <10 µM were determined.

File Name: Supplementary Data 3
Description: **Parasitological profiling of GHCDL hits**. Commercially available compounds from the Pf DGFA and Pf asexual assay hits were obtained and profiled across the parasite life-cycle in a variety of assays in *P. falciparum* and *P. berghei*.

File Name: Supplementary Data 4
Description: **Summary of Pf SMFA feed data**. Selected compounds were tested for their ability to prevent luciferase-expressing parasite transmission to the *A. stephensi* mosquito as estimated by mosquito luciferase intensity. Data presented here summarises all replicate feeds.

File Name: Supplementary Data 5
Description: **In vivo transmission-blocking activity of selected compounds.** Mice infected with *P. berghei* were treated with 50 mg kg^-1^ of selected GHCDL compounds by IP injection. The effect on asexual parasitemia, gametocytemia was recorded and *A. stephensi* mosquitoes were allowed to feed on the mice. Transmission-blocking efficacy was assessed by determining mosquito midgut oocyst burden.
